# Proceedings of the Iowa State Dental Society

**Published:** 1865-09

**Authors:** 


### Proceedings of Societies.

### PROCEEDINGS OF THE IOWA STATE DENTAL SOCIETY.

Held at Dubuque, Iowa, July 19th and 20th, 1865.

The Association being called to order by the President, Dr. L. C. Ingersoll, the following members were found to be present:

L. C. Ingersoll, Keokuk; Wm. O. Kulp, Jos. Hardeman, Muscatine; T. P. Smith, Tipton; A. P. Sayles, Lyons; H. S. Chase, Iowa City; R. D. Myers, Geo. W. Nichols, J. J. Severance, Wm. J. Adams, Davenport.

The President made a few pertinent introductory remarks, asking the Association to remember, that whether the number of its members be great or small, they are the representatives of the profession in Iowa; and urging them to conduct themselves in a manner worthy of the noble State which they represent.

The Secretary, Dr. Chase, then read the minutes of the preceding session of the Association, which were approved.

The following new members were admitted :—E. L. Clark, J. P. Porter, C. Poor, B. Rogers, J. W. King, Dubuque; Dr. Branch, Vinton; Wm. F. Gunkle, Davenport; J. S. Charles, Marion; Miss L. B. Hobbs, McGregor.

Dr. J. Taft, of Cincinnati, Dr. H. E. Peebles, of St. Louis, and several other Dentists, visiting from neighbouring States, were elected honorary members.

Other preliminary business being disposed of, an essay was read by Dr. Nichols on Mechanical Dentistry.

Dr. Branch rose to thank the Society for electing him an honorary member, and to say that the essay met his feelings very accurately. It touches upon points that are exceedingly important to the profession. While listening to it, I was rather congratulating myself that I was rather out of practice, and out of the profession, in these days. I called to mind the many times, during the twenty or twenty-five years I was in the profession, that I have been annoyed and distressed at the specimens of mechanical dentistry I have seen. And again, I was struck with chagrin at the idea their suggested, that the dentist must go shopping for business; that the price of our labor and our skill has depreciated till the price of a set of teeth has come to be at last about that of a pair of boots. This is seriously letting down a profession as honorable as any on the face of the earth; that ministers as much to the benefit of humanity, when rightly and properly practiced, as any profession can do. The dental profession owe it to themselves to sustain the prices and the quality of their operations, and to do everything in their power to discountenance and discourage whatever has a contrary tendency. It is in the power of dentists to make their own reputation. I like the suggestions of the essay in regard to the manner of inserting artificial teeth. It is an error to endeavor to do our work *too* nicely. Too often we endeavor to force nature to our ideas, instead of following up her ideas. Another thing, my own opinion is, notwithstanding the advantages—and they are many—of the present mode of inserting teeth on rubber base, the profession will one day come to condemn the whole thing as an injury to us. I am afraid of everything that cheapens the price.

Dr. BRANCH, of Vinton endorsed the ideas regarding the use of rubber for a cheap base. I live on the outskirts of the profession, where people are not so wise as they should be; *if they were*, no injury would result from the use of rubber. When men can buy a set of new teeth for the price of a pair of boots, it is hard work to induce them to expend twice as much, perhaps, for the sake of getting their old ones filled. I remember having a talk with an old gentleman, not many days ago, on this very matter; said he, "When I can get a new set of teeth for twenty dollars I am not going to spend forty or fifty dollars for filling the old ones." There it is, in a nutshell; you can see just how it stands. Under existing circumstances, perhaps, rubber is beneficial; but we want wise men, good men, honest men, to use it; to impress on the mind the superior advantages of preserving the natural organs. I consider this the principal use of dentists. The man who cannot save the natural teeth when he can get at them at the proper time, is not fit to be called a dentist. I would not depreciate the value of mechanical dentistry. It is a wise and beneficent arrangement that we *can* supply, to some extent, the place of the natural organs. But it is better to educate the people, till such measures shall be unnecessary; they should be taught the advantage of placing themselves and their families, from childhood, under the dentist's care, and thus anticipate the resort to mechanical dentistry.

Dr. Kulp.—Some things are necessary evils; and I look upon mechanical dentistry as one of them. Yet it is an important subject, for we all have more or less to do with it. Every true dentist feels it to be one of the things we are obliged to do, which we desire greatly to put away. We hate greatly to see a man without a limb, yet sometimes it is necessary to have it amputated to save the life of the owner. When this must be done, it is a blessed thing to have an artificial one, to take the place of the original one, in some measure; yet there is no person who would consider it a very pleasant thing to lose so valuable an assistant in life as a limb. I look upon mechanical dentistry in something the same light. There are persons without teeth, or with the natural teeth in such a condition that the individual is better off without them than with them. *When* gone, it is a blessed thing that we can make artificial teeth take the place of the natural ones, to some extent. All must feel grieved at the necessity of the operation; and none more than the true dentist. My idea has always been, that the true mission of the dentist is, to save the natural teeth. But there are some cases where we *must* use artificial dentures. When the teeth are actually gone, we look around to see how to supply the deficiency. Sometimes we are satisfied that the person can afford to pay for the very best; consequently we accommodate ourselves to his circumstances, and give him the best. But we know that the majority of those who are without teeth cannot afford to pay for the best operations; and were it not for the rubber base, or some such less costly article, they would be compelled to go without entirely. So, I am inclined to think that rubber is a blessing. I contend that it is possible to make, upon a rubber base, as good a set of teeth—as natural looking, as permanent, and that will do the patient as much good—as upon any other. The judgment of the dentist, and the patient's purse also, must decide. As rubber has been used by many, it is a curse; but the rubber is not in fault—it is the manner in which it has been used by those who are not honest men. It is just the same with filling teeth. Every day, almost, I am pained at finding teeth, pretended to be filled, where I could take an excavator, and run it through the plug to the bottom of the cavity. Filling teeth is in itself a blessing; but it is a curse when it is done in such a manner. Many things are blessings in their appropriate spheres, but out of them, curses. Food, if we need it, is a blessing; otherwise a curse—an injury to the physical system. So we ought to be considerate in our denunciations of any particular thing. As I have said, I look upon mechanical dentistry as upon surgery; sometimes a necessary operation, yet to be avoided if possible. We do not rejoice that the patient is in a condition where it is necessary to amputate a limb; but when the operation has been performed, we rejoice that we have the means of supplying the loss in some measure.

Dr. Taft.—Had two or three reasons for not wishing to speak. First, he didn't know much about mechanical dentistry: second, he preferred to hear other members of the Association. There were so many points, so many particulars, connected with the subjects presented by the essay, that unless one had decided beforehand upon what he would talk, it was rather difficult to speak. There is no one thing, perhaps, in which the majority of the profession commit a greater error, than in the removal of teeth—sound teeth, that ought not to be removed—for the purpose of inserting artificial teeth. It is a very common practice, when but *a few* teeth remain in the mouth, whatever their condition, to remove them. I was recently present at a conversation between several dentists, the most of whom advocated the idea of removing the teeth, when but few remained in the mouth, no matter what their condition might be. One operator said, if there were six or eight teeth in the anterior part of the lower jaw, and none others below, and none above, he always removed them, however healthy. Others, when there were only four or five, or six teeth left, advocated always removing them, regardless of their condition, in order to supply their loss with others. But I insist that when there are a few healthy teeth remaining in the mouth, they should remain, and not be removed for the purpose of inserting artificial dentures. We all know the difficulty of preparing a partial denture, especially when the teeth are left one here and another there. I know that such irregularities add greatly to the labor, and demand more skill of the dentist; but I know that in the worst cases of that kind, artificial dentures can be made that will be valuable. I contend that where there are but six teeth, for instance, left, even though they might be somewhat irregular, they should never be removed When there are even four or five, cuspids and bicuspids, in a healthy condition, the parts around them generally natural, and not absorbing down, let them remain. If there were but one cuspid, or one molar, I should remove it; unless there were teeth to antagonize with it in the lower jaw, and then let it remain. Of course, no rules can be laid down in regard to this matter, that can be applicable in all cases. Inserting artificial dentures, when the natural teeth remain, one here and another there, may be rather difficult; but we ought to cultivate ourselves in the art of doing difficult things. The practice of our profession is made up of things that are difficult of accomplishment in the beginning, but that become easy after awhile. Sometimes, when nearly all the natural teeth remain, it is best to remove them; because their condition is such that they will be only a source of annoyance to the patient, so long as they remain in the mouth; we must be governed by circumstances, and not by any fixed rule. I find this to be too much the case in our profession; the desire is to find some fixed rule by which to do everything; just as if we were working upon a piece of wood or iron. But in our profession no such invariable rules can be given. There are scarcely any two operations you will be called upon to perform that should be performed exactly alike; every case demands for its best treatment a mode of operation peculiar to itself. This thing of performing operations that are ostensibly similar, alike in every case, is simply empiricism. Yet we are too apt to do it; to work by one uniform rule; to wear a rut, as it were, and to keep in that rut all our lifetime; to make a set of teeth in all particulars as we have made hundreds of sets of teeth before—instead of acting with reference to the *particular case* on hand. Every case requires a mode of treatment peculiar to itself. I would especially impress upon the members of the profession the necessity of getting out of this routine way of doing things, and being governed in every case by the conditions present in the case.

Dr. PEEBLES—said he was not a speaker, nor a writer; but for the last thirty years he had been trying to *make his mark* in the profession. Thought Dr. Nichols, in his essay, took a very correct view of the subjects. I have said, on previous occasions, that I regarded the introduction of rubber and gutta-percha into the profession, for bases for artificial dentures, as a curse to the people and the profession. That requires some modification—yet I cannot take back what I have said. I feel no disposition to take it back. I find it is a matter of record in the American Dental Association, that I so regard it. Yet, as Dr. Kulp has very happily said, the introduction of the materials themselves may be a blessing, while the manner of using them turns them into a curse. For instance, the introduction of alcohol into the arts is a blessing, but the use of alcoholic drinks is a curse in the community.

I have advocated, for many years, the salvation of the natural organs of mastication. I exceedingly deprecate the wholesale extraction which many dentists seem to delight in. I love to extract teeth; and I am vain enough to think I can extract a tooth about as well as any man. Nevertheless, I greatly prefer to save every tooth I can. I have found this to be the fact in the community where I have been practicing —that before the introduction of these vegetable bases, these cheap bases, and the diminished expense of artificial dentures consequent upon their introduction, people were willing to pay more for the salvation of their natural teeth than they are now. And I have always regarded that as a criterion of the value people set upon their teeth—the willingness with which they pay for their preservation. When it cost two hundred dollars to have a double set of teeth put in, on a gold base, people desired to save their natural teeth, from the fact that it was costly getting artificial teeth. But now, when you ask them more for filling a few front teeth than it would cost to have them taken out and replaced by artificial ones, they can't see it in that light; nor in the light in which an educated dentist sees it. Therefore I consider the introduction of these cheap bases a curse to community. Another evil is, it has brought so many unqualified men into the profession; men who have sometimes no qualifications, beyond what the painter has given them in painting their sign; but they can "put up teeth," on rubber—as Dr. Nichols calls them, very appropriately—artificial "hoops"; can give them the "horseshoe bend," and put them into the mouth. Nevertheless, they are *teeth;* and the men who put them in, somehow sometimes manage to sustain a fair reputation. As I heard a lady say, " such a man is a good dentist; he put up a set of teeth for me that have lasted me for two years, and no break in them." Yet, if they had been mine I should have deemed them only a curse to me; but she was satisfied with them. Whatever tends to cheapen dentistry I consider a curse to the profession, and to the public. But I am happy to say, there are some who are not led off by these things; who are not led away to the wholesale destruction of their natural teeth.

I have no hope of living to the time when dentistry gets to be what it must become ultimately. Some of the young men here may live to see the day when there will be rare cases of putting in artificial teeth; but not in this generation; not in the coming half century. The time will come, I hope, when the first principles of dentistry shall be a matter of education in our common schools; when the children will be taught how many teeth they have, and how to take care of them. Now, a large majority of parents do not know. I tell young ladies they are not prepared to enter the marriage relation till they know how many teeth their children ought to have in their first set, and how many in the second. Many a sound tooth has been lost, because the parent or guardian, or even the attending physician, did not know that the six year molar was not to be shed and replaced by another. When people come to give a proper appreciation and attention to their teeth, they will give better teeth to their offspring than we have given to ours, or our parents gave to us. The time is coming when artificial dentures will be very little called for. Young men who are here may live to see the time when the dentist will not be a *tooth-maker*, but will let somebody else do that when it is necessary—as the surgeon does not *make* artificial limbs. I may be too sanguine in this matter, but I mean what I am saying.

I thank you for the honor of being made an honorary member of your Society. I came not here to talk, but to listen; to see the faces of these men, who are trying to elevate the standard of the profession of dental surgery. I am making a little tour of pleasure and observation, and to recuperate my waning physical powers; and expect to see the magnates of the profession before I return. I visited some of them last summer, much to my pleasure and profit. I went home a better man; a better dentist; I did better work. Young men, I am a student—still learning. I have learned more in the last twelve months, than in any twelve months of my life before; and I am now fifty-three years of age. Last August I felt myself giving out; I thought I would "wind up;" but I have changed my mind. I have taken a new lease of life; and now, by the grace of God—and the use of the mallet—I expect to stand it a good many years yet!

Dr. BRANCH, of Illinois.—Dr. Peebles has just expressed the idea I had; that in cheapening artificial dentures, we are holding out an inducement for people to loose their teeth, instead of saving them. But further than this. I contend that the rubber itself is absolutely a deleterious substance. It may be slow in its operation, as tea is said to be a slow poison; *very* slow, you know the old lady said, she had drinked it for eighty years, and it had not poisoned her yet. Rubber absorbs and wastes the foundation upon which it rests; the nerves are brought so near the surface, that an abnormal sensitiveness is produced; so that when cold water strikes the artificial teeth, it produces an unpleasant sensation, as in the natural teeth, and they really *ache*, like the natural teeth. I have noticed a rapid and serious absorption take place. I am afraid of the substance itself; I believe it will bear watching. There is nothing safer than gold; but there are inducements to using other substances, that are paramount, in too many instances. Almost any man can insert a rubber denture without any difficulty, while to fit a gold plate, he has something more to do; and those who have never worked in gold would find it out. Yet I believe gold can be made as useful as rubber, so far as practicability is concerned.

It is the business of our profession to educate the public, rather than come down to their ideas of what we ought to do. Let them understand that we are the ones to tell them what to do, and not them to dictate to us what to do. When they say, "I can get such a man to do so and-so," we are too apt to say, "Well, then, *I'll* do it." Better say, "you must get others to spoil your teeth, if you insist upon having them spoiled, for I won't do it."

Dr. PEEBLES.—Dr. Branch's last remarks reminds me of an incident that occurred within my experience, not long since. A lady called upon my friend, Dr. Morrison, of St. Louis, to have four teeth extracted; he politely told her she ought to have them filled. Said she, "I did not come to have you tell me what to do, but to tell you what to do." The conclusion of the matter was, he refused to extract them, and the lady came to me. Of course, I did not know that she had just left his office. Said I to her, "you do very wrong to have those teeth extracted." "But," said she, "one of them is paining me." I told her that I could relieve the pain and save the tooth; that it would cost her more than to extract them, but that it would be a great deal better for her. Said she, "I did not come to be dictated to, but to dictate;" and in the conversation that followed, I learned she had already been to one dentist, who had refused to extract the teeth, and I became more positive in my refusal. I told her I would be doing her an injury, and would certainly be doing my profession an injury. She fretted considerably; but finally inquired how much it would cost. I told her. At first she objected to the price. Said I, "Madame, would you have one of your good teeth taken out for twenty-five dollars?" She said, of course she would not. I told her I could make either of these last her as long as any other in her mouth. So we reasoned together; and at last I filled four teeth for her, for sixty-five dollars; and it was paid as cheerfully as she ever paid a debt in her life.

Now, gentlemen, we must not be too pliable in the hands of the public. We must not do what we conscientiously believe to be wrong. When I am sure that I can save a tooth, I always refuse to take it out; and I know I have lost quite a number of cases in that way. If, under such circumstances, the patient gets up and leaves my chair, he can do it. It is the same way with regard to fitting new dentures into mouths where the teeth are partly gone, and some that I know ought to be left. I always dictate to my patient what I know ought to be done. I leave it to their conscience whether they will submit to my dictation or not; but almost always, where the operator is decided, conscientious and honest, they will feel that he is right, and submit to his operations. Sometimes I get fifteen or twenty dollars for saving a tooth, where, according to our professional rules, I could extract it and insert a new one for eight. I have also received for saving six front teeth, and replacing the others, more than the price of making a full upper and lower set, which the patient at first desired. I find cases similar to these every day. All that is necessary is, for the dentist to say "no;" and convince the patient, by his frankness and earnestness, that he knows what ought to be done.

Dr. Gunkle.—Coincided with the views expressed by the members generally, with respect to rubber bases for teeth. In my opinion, the introduction of rubber has been a great injury to the people. Since then, more good teeth have been sacrificed than all the dentists in the country have saved. Another objection he has to rubber is, that teeth upon a rubber base lack expression; they lack naturalness; they appear to be an imitation; they make the wearer look like a "wooden man." I never saw a set of teeth on rubber that came up to my ideas; there is something about them that *looks* artificial. If dentists did not confine themselves so much to blocks, there might be an improvement. I don't know what better we could do than to get up indignation meetings, and put the thing down. For my part, I would be willing to do it. In going into the majority of dentist's offices, half the teeth inserted are full sets, or upper sets; and probably half the patients are aged between eighteen and forty. I do not think there ought to be, on an average, one full set in a hundred. I do not believe in the extraction of even *one* healthy tooth, unless in case of absorption of alveolar process. If the canines, upper and lower, antagonize, and keep the mouth in place, I believe in allowing them to remain. I do not believe in extracting *any* healthy tooth, if an artificial denture can be made.

Dr. Nichols.—I look upon rubber, as the rest of you do, as an injury, taking into cansideration its general influence upon the people, causing them to sacrifice their natural teeth. But there are circumstances under which I would use it. Many persons who needed teeth, could not afford gold plate or continuous gum work. I certainly would not give them silver, for I consider that far more objectionable than rubber itself. In such case I would put up a rubber set; and with the same teeth I think I could give equal expression as if I fastened them on a gold plate. Fastening teeth to gold plate with rubber attachment, I consider a very good method.

Dr. Taft.—In reference to rubber, I felt for a long time just as has been expressed by some others here. Afterwards I came to the conclusion that I had been looking at one side of the question too much. I had been disposed to attach blame to the material, which did not really belong to it. Much that has been said against it is true; and so is much that has been said in favor of it. It is true that it has brought a host of men into the practice of dentistry, who are not dentists at all; merely tooth-makers; men who ought not to be acknowledged as dentists; who ought not to be acknowledged *in any sense whatever*, by members of the profession. A great many have climbed over the wall, torn through the hedge, got into the profession somehow, illegitimately, and are doing that kind of work. There were some of them before; but more now, for this method is more easily obtained than any ever used before. Many who are using the rubber in an objectionable manner, were it not in use, would be using something else in a manner just as objectionable. If rubber is not injurious—and I do not regard it so, at all, in itself—our invectives should be not against *it*, but against the reprehensible manner of using it. Rubber is made into artificial dentures in such a way as to injure community, both directly and indirectly, and to operate against the interests of the profession. All that you can say about the injury done to the people and the profession is true. Yet the rubber is a good thing—a very good thing; and in many cases I regard it as the very best material upon which to set artificial teeth. It has wrought a great injury in the minds of the people; at least of that class of people who do not recognize the value of their teeth, and the importance of preserving them. Knowing they can get artificial teeth so cheaply, they become careless of their natural organs. When you can take out teeth without pain, (by means of chloroform, ether, or nitrous oxide gas,) and put them in without charge, you will find the great mass of the people doing just this thing; getting rid of the teeth that were paining them, and having inserted others that will not. You cannot stem that tide by a direct effort; you have got to make a "flank movement" on it; you must educate the people. The mass of the present generation, I expect, will go down to their graves in ignorance and darkness; but we can educate *some*; and let us do that. This is our great duty now—to inform the people, by conversation, by written essays, by public speeches, by the presentation of the truth in any and every manner we can. Let them see what is for their real interests, and these evils will correct themselves. Let every true dentist place as great a distance between himself and the mere "tooth-maker," as possible; and draw the line of demarkation as distinctly as possible, so that he who runs may read. I have known many very happy instances of that kind; of persons who were in total ignorance of the value of their teeth, becoming enlightened upon the subject, till they are now the best patients we have. It is not practicable to say this or that man shall not practice dentistry. Our only alternative is, to educate the people till they shall be able to distinguish a good dentist from a poor one, and act accordingly.

Dr. KULP.—Sometimes we help these charletans by denouncing something they use that is really beneficial; perhaps we have done this with regard to rubber. The enemies of rubber have denounced it in wholesale, with all the epithets that could be applied to it; yet these men have used it, and have given perfect satisfaction with it. And the very fact that you have denounced it, and yet it has proved a good thing in their hands, has put you down, and put them up; put gold down, and put rubber up. It is time we made the "flank movement" suggested; give rubber its due, and expend our denunciations on the men who misuse it.

Dr. SEVERANCE.—Whatever we say about the rubber, I think the price of mechanical operations will be regulated pretty nearly by their actual value. If a man send out a good piece of mechanism, he will be able to get a good price for it; if a poor piece, the contrary. A poor workman, seeking to extend his practice, will be apt to offer his work at a reduced price. I have not had a great deal of experience, but this has been my experience, so far as it has gone.

Dr. INGERSOLL.—(Surrendering the chair to the Secretary, Dr. Chase.) I do not like to have the discussion of this subject stop where it is. I do not think the remarks made so far, have expressed clearly the sense of the profession. Before we condemn a thing, we ought to take into consideration not only its disadvantages, but its advantages. Let us make a supposition. We know that gas is the best light there is for ordinary illuminating purposes. Now, when coal oil was discovered, supposing the gas manufacturers had gathered together and protested against the use of coal oil, because it would cheapen light; they make no other objection. That has been the main drift of the rubber discussion, so far. Let us look for a moment at the value of rubber, as a base for artificial teeth. Certain requisites are essential. First, a perfect fit. I think it is universally acknowledged, that with rubber we can secure a *better* fit than with any thing ever used. Another thing to be sought after is, the restoration, not of the teeth alone, but of the voice; a thing passed by too often, and generally not looked upon as pertaining to dentistry; but we all know what a dilapidated condition the voice is in, when the teeth are gone; and it should be a part of the business of the dentist to restore it. With gold and silver, and even with platina, this is often very imperfectly done; indeed, often scarcely improved. Here there is a vast advantage in rubber; the restoration of the voice is almost perfect. You restore the natural form of the mouth more perfectly than is possible with any other substance. True, the voice is *formed* lower down in the larynx; but it is *moulded*, as it were, in the mouth. Another thing is, the expression of countenance. This depends not alone upon the teeth, but upon the proper position of the lips; and this can be secured with rubber more certainly and perfectly than with any thing else. Expression of countenance is something very valuable; no one likes to lose his identity; and if you can preserve it by your artificial substitutes, there is a great thing gained. I contend that anything having these vast advantages should not be condemned at wholesale. In many cases I should put in rubber in preference to any thing else. As to the cheapening of it, we begin at the wrong end of the matter; the rubber is not to blame for being less costly than gold or platina; we ought to condemn the man who uses it. If A, B and C, unqualified men, charletans, are cheapening rubber, let us study artistical dentistry, so as to make a perfect fit, restore the voice, preserve the expression, and then we can keep our prices up to a proper standard, in spite of the lower prices at which unqualified and unprincipled charletans may offer their wares. Then, too, the dental profession would learn to prize rubber as the grandest invention we have had for many years.

Dr. INGERSOLL next read an essay on DENTAL ETIQUETTE

Dr. KULP.—This subject ought not to be passed over silently. It is one we all ought to feel considerable interest in, for it is one too much neglected by the dental profession. As a general thing, we take it for granted that we owe nothing to a professional brother; that we each have our own row to hoe, and we must hoe it the best way we can: at least, such has been our action towards each other. But the time is coming when we cannot have every thing our own way. A different material—better men—are entering the profession. In dealing with the public, in taking students, in all our transactions, we ought to have the golden rule in view. To begin with, we must be honest men. I doubt whether a dishonest man can make a good dentist. There is a great deal of selfishness in the profession. I do not know as there is more in ours than any other; still there is a great deal. I have more opportuuity of finding out this sort of spirit, occupying the position that I do, of Corresponding Secretary of this organization. It seems almost impossible for some men, good at heart, I verily believe, to think or care for any one's interest but their own. They can fight their own way, and that is all they care for; at least, that is the impression conveyed to younger dentists by many older ones. I know that every advantage possible was taken of me by older dentists; they appeared to do every thing they could to keep me down, when they knew my only desire was to learn, to climb the ladder of professional perfection. (I am getting pretty heavy, and may be they were afraid I would break the ladder down!) I would be glad to have each member of the profession come to me, and in an unceremonious and friendly way, tell me just what he thinks of me, and my work, and how I can better it. We must get rid of this selfishness and jealousy. I hope the time will soon come when every dentist in Iowa will subscribe to a stringent rule of etiquette—a code of ethics—drawn up by this organization; and consider themselves bound by its rules in this respect, as in any thing else. We all agree that this organization has elevated the professional standard; has made us better dentists, and better men. I can see the advancement in the members of the profession; their hearts are growing bigger. I know, in my own case, I love my profession better than ever before; I am prouder of it than ever before. I am jealous for the honor of my profession, and will zealously labor for its advancement, even at my own personal sacrifice, if necessary. When we have this love for our profession, we have the right kind of seed in our hearts, and can feel it springing up.

We never ought to say any thing against any member of the profession, in order to break him down, and build ourselves up. When a man in the profession insists on doing things in a wrong way, in a way that disgraces the profession, the interests of the profession demand that such a man be exposed.

Dr. Hardeman,—The essay breathes a just spirit, and the sentiments expressed are those to which every dentist who is a lover of virtue and good breeding will of course subscribe. In my own experience I have not had a great deal of trouble, because I do not repeat what I hear, or trouble myself about what others say. Unpleasant and almost revengeful feelings are sometimes created by a patient going from one office to another, and carrying reports that may be true, or partly true, and may be not; may be entirely false. The best way is, not to listen to them. Such things should not move us. One great annoyance to dentists in contiguous districts, or in towns where there are several together, is to have persons come telling how cheaply some one else will do their work for them; this is done in order to "drive a trade" with advantage to themselves. The only thing for an honorable man to do, is to take a direct straight-forward course, do his work well, charge fair prices, and especially never meddle with a patient belonging to another dentist. There need not be so much difficulty as there is; but there is a sort of morbid sensitiveness between members of the profession—and there is apt to be between the different members of any profession—that creates much of the trouble complained of.

Dr. Nichols.—After being sufficiently careful about the recognition of a practitioner as a dentist, then adopt the rule to love him in spite of fate, and it will be all right!

Dr. Branch, of Illinois.—The first speaker hit the nail on the head exactly, in regard to honesty. If there was ever a place where an honest man was required, it is by the side of the dental chair. I have said a thousand times, I believe there is no place in the world where dishonesty has so large a field to operate in, as in dental practice. Our patients may be satisfied with our operations for years, and yet we may have been very far from being faithful to them in our operations.

So far as trouble between professional brethren is concerned, I believe a share of it is unnecessary. If a man's heart is charged with high-toned honesty and integrity, he will be slow to hear, and slow to believe, any wrong of his fellow practitioner. The fact is, two-thirds of the trouble there is among dentists is made by their patients. We ought to remember that our patients do with us as with dry-goods shops; they depreciate the value of an article, and try to get it at a lower price by saying—"I can get this at such a price in some other store." So they go from one to another dentist, telling how low they can get work done somewhere else. They may not mean to lie, but they will manage to give it a twist that is not exactly true after all. One case I might mention as an illustration, to show what patients will do. Some twenty-five years ago I had a patient for whom I filled a tooth; a large molar. That was before we had learned to treat these ulcerated teeth. He asked me to fill it. I told him I could fill it, but it might not answer him any purpose at all. I could make my work stay there, but like as not, in a month or two he might wake up some night with a terrible toothache; then said I, the filling will have come out; and if you can dig it out, do so. Well, just about a month afterwards, the man woke up one night with a terrible toothache. He dug out the filling I had put in, and for a while it felt a little easier. But in a few weeks he came for me to extract the tooth. As I was just getting hold of the tooth, he grabbed my forceps, and broke the tooth off close to the gum. The patient went about the country saying, "Dr. Branch filled a tooth for me, and the filling hadn't been in a month before I had to dig it out, because it hurt me so; and then I went to him to have him pull it, and he broke it off square with the gum." And that was the end of the story. He told no lie; he stated facts, as far as he went. So our patients go, from one dentist to another, with stories that have as much foundation as that, and generally no more. They tell so much of the truth as suits them, and keep out of sight other circumstances that might make the matter present a very different aspect.

Dr. Branch of Vinton.—During the past two or three months I have been in a good many dental offices; I have conversed with regard to this matter of mutual intercourse between dentists; and I find there has been more or less competition or bidding in prices. After all this matter of etiquette narrows down to a matter of dollars and cents. Violations of professional etiquette are generally prompted by pecuniary interest. We have a rate of prices agreed upon; and yet there appears to be a lack of confidence in each other, in regard to adhering to the prices established. Dentists appear to be suspicious of each other; and I often hear language something like this: "Well, if Dr. So-and-so tries to underbid, I think I can operate just as low as he can." Is this the correct principle for an honest, high-minded dentist to act upon? If our competitors act dishonorably, (for we all *are* competitors in one way or another, directly or indirectly,) it is the part of a man of principle not to act dishonorable also, but to sustain himself as long as he can, honorably; and if the place will not support him, let him go elsewhere.

Dr Severance.—The subject under consideration is one of immense importance to us, as a profession, and especially to those members of the profession who are located two or more in the same town. A union price list, if honestly adhered to, is a great benefit. It is calculated to do away with much heart-burning and jealousy; to bring members of the profession together on terms of equality. Fearing no competition other than that which is legitimate, when they meet they are willing to exchange thoughts on dental subjects. In our city (Davenport) we have adopted a price-list; we have worked under it since the convention met there; and from that time we have been more friendly, more social with each other; the little petty jealousies that were formerly known among us have passed away. Yet I presume I have heard remarks, and heard *of* remarks, that if I had listened to, and repeated, might have made trouble. Sometimes a patient comes into our office and asks our price for an operation; we tell him; he tries to beat us down; we refer him to our price-list; "O, yes," says he, "I know you keep that up to scare folks with; but you don't pretend to abide by it; I don't care anything about the prices you have marked there —just tell me what you will do it for!" We refuse to do work for less than the prices agreed upon, and our patient appears surprised at our audacity in sticking to our figures. "That will do to talk," he says, "but we all know some dentists will do their work cheaper than others." But I believe, in our city, such persons have invariably failed to gain their point when they wished to get work done cheaper than our established rates. At least, I have no proof that any member of the profession has violated his word. We have worked in harmony. And wherever that system is introduced, harmony must prevail.

Dr Taft.—One think not to be overlooked, in regard to this matter, is its influence on the public. The public effect of the treatment of professional brethern towards each other, in any department of medical practice, is the same. When professional men are hostile one to another, the effects upon the people are miserable. Where you find the fellow members of the profession, in any community, speaking disrespectfully of each other, it neutralizes the confidence the people would otherwise have in them. If they speak disparagingly of one another, calling each other ignorant, dishonest, &c., the people take it for granted it is so. If the members of a profession do not sustain the honor and reputation, they can not expect outsiders to do it. If they treat each other as they should, do all they can in behalf of each other, it strengthens the confidence of the people in them. When dentists, or medical men in any department, make common cause and sustain each other, in sustaining others, each one sustains himself, and they can command the highest possible respect from the community. When one dentist speaks against another to a patient, he lowers *himself* in the estimation of that patient. If A *knows* B to be dishonest, or a bungler in his operations, it only injures himself for *him* to speak of it. Of course, one ought never to sustain another in an obviously, wrong and wicked course. But if a dentist knows that a professional brother is doing as well as he can, even though that may be very poor, he should sustain him and hold him up, as a matter of self-preservation, and the preservation of the honor of his profession. There is no city in the Union where dentists stand better with the people, or one with another, than at St. Louis; there the profession does just about what it pleases. And this is because they sustain each other. There they have the best operators in the country, because there is a constant interchange of ideas. There are many other places where the same thing has been done to some extent. In our city (Cincinnati,) no dentist will receive a report concerning another dentist, from any person Things have been reportcd that had a tendency to make trouble; but we always go first to the one of whom it is reported, and everything is cleared away at the time, generally to the entire satisfaction of all. This is better than to let such things rankle in our hearts, and to harbor a feeling of jealousy and dissatisfaction. It is true that the most of the ill feelings that arise between dentists, has its origin with their patients. As for me, I do not want a patient who goes shopping with his custom from one dentist to another. I want patients who come to me because they have confidence in my ability, integrity and honesty. One who has not, I do not want to come to me at all. And now our patients have learned better than to go with their reports from one to another of the profession. I have not heard of a report calculated to awaken ill feelings being carried from one dentist to another in our city in a year; in fact, it is so long since that I can not remember when. There is a perfect understanding between the members of the profession. If there is anything one of them does not fully understand, he feels at perfect liberty to ask another. If there is one that fails to perform some operation well, the next will show him, and help him, till he can do as well as anybody else. In that way we have made some good operators of men who were mere bunglers four years ago. I tell you it is for your own interests to sustain your professional brethren everywhere. It will result in good every way to yourselves.

Dr. Peebles.—Just here I want to relate a little incident. Not long since a peculiar case came into my office for treatment. On examining the case, I felt a deep interest in it, I wanted my professional brethern to see it. I took five cards, on each of which I wrote simply my name, and the words, "Come now!" and sent by my office-boy, to the five different dentists whose offices were situated nearest mine. In twenty minutes the whole five were there. One said, "I come now;" and assured me he had left a lady patient in the operating chair, with her mouth propped open, for he knew when Peebles sent for him, there was something of interest to be seen. That is the feeling between the different members of the profession in St. Louis. Any man who does not fully understand how to perform an operation, or wishes to advise with those more experienced, feels perfectly free to send for one or more of his professional brethern, who is willing come and advise or assist. They say to the patient, "Now, we wanted you to have the benefit of all the skill we could bring to bear upon your case;" and that dispels any feeling on his part that the dentist he has called on didn't know what to do, and had to call in help.

Dr. King. Mentioned the fact that a year or two ago a dentist (not one of these present) came to the place where he was, and took special pains to advertise that he would put in full sets of teeth for thirty six dollars—the same Dr. K. was charging sixty dollars for, thinking that a fair price; and stated some other instances that had come within the range of his experience of similar violations of honorable conduct and professional etiquettes; and gave this as one reason of his delay in becoming a member of the association. He hoped the association would live up to the price-list already published, or if there were any faults in it, remedy them. He considered this matter of professional etiquette one of great importance to the profession.

Dr. Chase read an essay on "Lancing the gums in extraction of teeth," (for which see another page.)—Ed.

Dr. Clark, being called upon, said he came to listen, and not to speak. I can not conceive how many teeth, roots, &c., could be taken out without lancing. As one of the previous speakers has said, I think I can extract teeth as well as any one; I surely ought to, for I have been in the daily practice of it for twenty-four years. In some case it is not necessary to go through the process of lancing the gums; the teeth do not adhere so closely but they can be removed without it. But in a majority of cases I believe lancing useful, and in many cases absolutely necessary.

Dr. Smith.—I fully endorse the sentiments of the essay. I think the lancet very useful: many teeth can not be safely extracted without it. Many could be extracted without it, but with it, with more ease and less pain to the patient. We should take into consideration the patients' feelings, as well as the benefit of the profession. When I am preparing the mouth for an artificial denture, and find one or two sound teeth to be extracted, I scarcely ever cut the gum, except in case of roots, or I find a cartilaginous formation. Then the pain would be much less, and the operation much more nice. In inflammation around the roots, there is a strong tendency to adhesion of the gums, and then I deem the lancet indispensible. I keep my forceps with their beaks sharpened down and ready for use; if accidentally dulled, I repair immediately, and never use them except when they are in good condition. I never use a lancet without examining the edge. I want my lancet very sharp, using a fine hone—an Arkansas hone I deem the best—and harness leather for strcps. I never thought what proportion of cases could be got along with without using the lancet; not over one in twenty, I should think.

Miss Hobbs.—My experience has not been very large, as compared with that of old practitioners. I came to hear, and not to be heard; I did not expect to teach, but to learn. I certainly find the lancet very necessary. I may have to use it more than you gentlemen, who have stronger hands. I find pulling teeth more than I have strength to perform, unless I take every advantage possible. Three-fourths of my operations are the extraction of roots, and I have, of course, to use the lancet. I find my patients complain less when I use the lancet than when I do not. I use the lancet in almost every case.

Dr. Hardeman.—I fully endorse the sentiments of the essay, and also the remarks that have been made. I presume I do not use the lancet as often as many. As regards the sharpness or bluntness of the lancet, I leave that to the operator. Some prefer an instrument that has not a very sharp edge, but with sufficient taper and polish to strip the gum from the teeth by mere pressure. When there exists a predisposition to hemorrhage, sharpness of the lancet may be dangerous. When my patient is under the influence of anesthetics, I never use the lancet, but the points of the forceps instead; it economises time, which is very valuable under such circumstances; especially when there are a number of teeth to be extracted. I push my forceps down, close to the tooth, till I can feel the alveolar process with them. In most other cases I use the lancet, cutting as close to the tooth as possible.

Dr. Kulp.—In my experience I have used the lancet almost always. Sometimes with a timid patient, who would rather stand considerable pain than have the lancet used, I take my forceps, put them down to the neck of the tooth, and with a rotary motion separate the gum and tooth, and take out the tooth; in that way I have extracted six without taking a lancet to them. I always use the lancet, except under the circumstances I have described; and then, I contend, I hurt my patient worse than when I use the lancet. I tried the other method for awhile, about a year and a half ago, but not to my satisfaction. I sometimes made sad work of it. One time I carried away a piece of the gum as large as the end of my finger. Some men, I know, never use the lancet, or say they never do; but I fancy this is done to make themselves conspicuous, or have it understood they are ahead of everybody else in their style of doing things. Some men get on a hobby and ride it to death. Those who say they never use the lancet, perhaps if we looked over their case of instruments we would find one somewhere.

I am no public speaker; but I can appreciate what others say as well as any man you ever saw. I never lance a gum when there is morbid irritation about the teeth. With a healthy gum I always use the lancet. A sharp, fine, hook-pointed lancet causes less pain than anything else you can use. A sharp burnisher, which does not cause much bleeding, can be used to remove the periosteum up to the alveolus. Where you use the lancet, it generally runs off into the nervous tissue and produces pain. A healthy gum should always be separated before you attempt to extract a tooth.

Dr. Severance.—It seems to me that one thing has been overlooked, Preceeding speakers have urged the necessity of using the lancet in extracting roots; and they have been talking on the supposition that these roots are to be extracted by forceps. I generally use an elevator or hook. I don't see how one *could* use this instrument without he did separate the gums. I use a lancet that is shaped a good deal like a sickle. I first make an incision from about a quarter of an inch below the connection of the gum with the tooth, up the center of the root, and pass it around forward; then around towards the back part of the tooth, or the inside, in the same way. Formerly, I did extract with forceps, and then I frequently did not use any lancet. Sometimes I took out the alveolus and root too. Sometimes I didn't get either. More recently I use the elevator and hook—the latter principally for the back teeth.

Dr. King.—I must confess I do not know why, but not for the sake of being odd, or getting ahead of other practitioners, but it is very rarely that I use the lancet, except where the root is entirely obscured by the growth of tissue. When I can get a fair hold of the tooth, it seems to me that forcing the lancet around hurts as much as crowding the forceps on; and then crowding the forceps on is so much more pain. In removing fangs, whether the lancet is used or not, one thing I have observed, that ought to be remembered to advantage. A root, where the tissues around it have become absorbed, is not held in place by any pressure of the alveolus, or tissue surrounding it; it is merely obscured by the gum; it lies there in the jaw, like "a stick in the mud;" by "humoring" it a little, and being very careful with it, the slightest hold possible will frequently remove it with very little effort on the part of the operator or pain to the patient. But the root is often a mere shell, very tender and brittle, and if it be crowded upon, to get what is called a "firm hold," the little bony sliver will be broken off, and all opportunity for getting hold of the fang is gone. I speak more particularly of fangs entirely burried in the tissues.

Dr. Chase read an essay on "Lancing the gums of Infants."

Dr. Taft, being called upon, remarked that this was an important subject, concerning which considerable diversity of opinion existed; a subject which should be far better understood than it is. The methods of relieving the sufferings that arise from difficult dentition are various, because based upon different theories in regard to the origin of these difficulties. I suppose that in many cases the lancet is resorted to unnecessarily; but again, in many other cases, it is the best thing that can be done. It will frequently produce immediate relief. Wherever the distended condition of the gums and an engorgement of the vessels in the parts, is the cause of a great portion of the pain, the sufferings of the little patient are generally promptly relieved by the use of the lancet. But pain is produced during dentition from other causes. The development of the growing organ causes pain. The pressure of the coming tooth against the gum causes pain. But I suppose the pain is caused, not so much by the pressure of the tooth against the gum, as by the general irritation occasioned in the system, and the want of preparation for the passage of the teeth through the gum, from the absorption not being sufficient to accommodate the parts to the exit of the teeth. I infer this from the extent of the irritation; it is not local, but general, extending through the entire system of the child. There are many courses of treatment I think well to resort to. Lancing the gums, to relieve the vessels of the blood accumulated in them, is one method, and in many cases proves very efficacious; but not in all. Sometimes it seems to produce no good results, even for the time being. Sometimes when temporary relief is obtained, the wound is healed by first intention, closes up, and, in a few hours, the patient is as bad as ever; and repeated cutting is necessary. Where you find cutting the gums will relieve the suffering, it is better to perform it, even if it has to be repeated every forty-eight hours. Generally it will be unnecessary to resort to it more than once or twice. The indications for cutting are, when the irritation is confined chiefly to the contiguous parts. When the irritation extends through the nervous system, and other organs are brought into sympathy, something else is indicated; or at least something more.

In lancing gums to assist dentition, or to relieve irritation accompanying it, the *manner* of doing it will be determined somewhat by the teeth. If incisors, cut directly over the tooth, upon a line with its edge, carrying a lancet to the tooth, but not hard enough to injure it. If a molar or bicuspid make two incisions, one at right angles with the other, down to the crown of the tooth, raising the flapes made by the incision. If the wound shows a disposition to close, put a few fibres of cotton under the angles of the wound; it will occasion a little ulceration, or sloughing, but that does not matter. Caution is to be observed with reference to a hemorrhagic diathesis. There is sometimes serious difficulty, with children, in managing a case of this kind. There are numerous cases on record of children dying from hemorrhage, occasioned by the use of the lancet to assist dentition. If a hemorrhagic predisposition is known to exist in the family, lancing the gums becomes a question of serious consideration. I have seen cases where I thought that a small incision into the gums would relieve the child; but I dare not do it.

As a general rule, then, I would not hesitate to use the lancet in cases of difficult dentition, especially if the difficulty was local. Where the system is affected, other treatment is necessary.

(*To be continued*)

